# Transdermal Immunization of Elastic Liposome-Laden Recombinant Chimeric Fusion Protein of *P. falciparum* (*Pf*MSP-Fu_24_) Mounts Protective Immune Response

**DOI:** 10.3390/nano11020406

**Published:** 2021-02-05

**Authors:** Ramesh Chaudhari, Nikunj Tandel, Kiran Sahu, Sushmita Negi, Hilal Bashir, Arzu Rupareliya, Ravi PN Mishra, Sarat K. Dalai, Rajeev K. Tyagi

**Affiliations:** 1 Institute of Science, Nirma University, Ahmedabad 382481, Gujarat, India; patelramesh216@gmail.com (R.C.); nikunj.tandel@nirmauni.ac.in (N.T.); arzur170@gmail.com (A.R.); sarat.dalai@nirmauni.ac.in (S.K.D.); 2 Division of Cell Biology and Immunology, Biomedical Parasitology and Nano-Immunology Lab., CSIR-Institute of Microbial Technology (IMTECH), Sec-39A, Chandigarh 160036, India; kirankumariroyal@gmail.com (K.S.); negi.sush3@gmail.com (S.N.); 3 Division of Cell Biology and Immunology, CSIR-Institute of Microbial Technology (IMTECH), Sec-39A, Chandigarh 160036, India; hilalbashir65@gmail.com; 4 BERPDC Department, CSIR-Institute of Microbial Technology, Sector 39A, Chandigarh 160036, India; ravi.mishra@imtech.res.in

**Keywords:** malaria, elastic liposomes, *Pf*MSP-Fu_24_, humoral and cellular immunity, vaccine

## Abstract

Transdermal immunization exhibits poor immunogenic responses due to poor permeability of antigens through the skin. Elastic liposomes, the ultradeformable nanoscale lipid vesicles, overcome the permeability issues and prove a versatile nanocarrier for transcutaneous delivery of protein, peptide, and nucleic acid antigens. Elastic liposome-mediated subcutaneous delivery of chimeric fusion protein (*Pf*MSP-Fu_24_) of *Plasmodium falciparum* exhibited improved immunogenic responses. Elastic liposomes-mediated immunization of *Pf*MSP-Fu_24_ conferred immunity to the asexual blood-stage infection. Present study is an attempt to compare the protective immune response mounted by the *Pf*MSP-Fu_24_ upon administered through transdermal and intramuscular routes. Humoral and cell-mediated immune (CMI) response elicited by topical and intramuscularly administered *Pf*MSP-Fu_24_-laden elastic liposomes (EL-*Pf*MSP-Fu_24_) were compared and normalized with the vehicle control. Sizeable immune responses were seen with the transcutaneously immunized EL-*Pf*MSP-Fu_24_ and compared with those elicited with intramuscularly administered antigen. Our results show significant IgG isotype subclass (IgG1and IgG3) response of specific antibody levels as well as cell-mediated immunity (CMI) activating factor (IFN-γ), a crucial player in conferring resistance to blood-stage malaria in mice receiving EL-*Pf*MSP-Fu_24_ through transdermal route as compared to the intramuscularly administered formulation. Heightened immune response obtained by the vaccination of EL-*Pf*MSP-Fu_24_ was complemented by the quantification of the transcript (mRNA) levels cell-mediated (IFN-γ, IL-4), and regulatory immune response (IL-10) in the lymph nodes and spleen. Collectively, elastic liposomes prove their immune-adjuvant property as they evoke sizeable and perdurable immune response against *Pf*MSP-Fu_24_ and justify its potential for the improved vaccine delivery to inducing both humoral and CM immune response.

## 1. Introduction

A major bottleneck in developing an effective vaccine for infectious diseases is suboptimal or no elicitation of non adaptive immune responses and subsequent potent stimulation of perdurable and long-lasting adaptive immune responses. Therefore, appropriately selected adjuvants and delivery systems are required to mount sizeable immune responses *en route* to the vaccine development [1]. Intramuscular (IM) route of vaccine administration has been prominently used for the majority of vaccines and only a few vaccines are administered through transdermal (TD) route. The suitability of IM route has been established due to its well tolerability, and only minimum chances of adverse risk at the site of injection were observed [2]. However, needle phobia, need of aseptic administration, and requirement of trained personnel advocates for the noninvasive transdermal route over the invasive route of vaccination since it avoids the systemic side effects, maintains uniform blood levels, and increases patient compliance [3]. Therefore, transdermal route of delivering antigens through skin has been considered as an immuno-competent site to amplify the antigen-specific immune responses. In a study, significantly heightened immune responses were quantified when yellow fever virus vaccine or influenza [4], Hepatitis B [5], and *P. falciparum* [3] vaccine candidates were administered through TD route.

Furthermore, transdermal immunization has been shown to reduce dose of antigen while maintaining the greater immune responses. Recently, intradermal administration of mRNA-based vaccine was shown to efficiently activate the antigen-presenting cells (APCs) at the site of injection. It also induced transiently higher vaccine-specific T cell responses and antibody (Ab) titers as compared to those seen with intramuscular vaccination [6,7].

*P. falciparum* is the leading cause of malaria infections worldwide, contributing to the greater rate of malaria-associated morbidities and mortalities in sub-Saharan Africa [8,9]. There are a number of approaches to malaria vaccine development based on attenuated sporozoite and synthetic and recombinant immunogenic peptide, but these suffer from drawbacks of being potentially unsafe and developing short-lived species and stage-specific immunity [10,11,12]. These approaches induce weak immunogenic response for conferring protection against *P. falciparum*. Moreover, complexity of *Plasmodium* parasites, antigenic polymorphism, species and stage-specific variability poses challenges for maintaining long-lived immune responses [13] against *P. falciparum* [14,15].

The erythrocytic merozoite invasion process is one of the promising targets for developing vaccine against malaria, and merozoite surface protein-1 (MSP-1), a polypeptide of 190–230 kDa, has been widely considered as a component of malaria vaccine [16,17]. MSP-1 undergoes proteolytic cleavage and produces four fragments of variable molecular weights (83, 28–30, 38–45, and 42 kDa) at the time of rupturing of schizonts and prior to the rupturing of infected erythrocyte at the end of 48 h replicative cycle to release merozoites [18].Carboxy terminal, cysteine rich, 42 kDa (MSP-1_42_), is further processed to yield a 19-kDa fragment (MSP-1_19_) that remains associated with merozoites. The immunogenic potential of MSP-1_19_ and MSP-1_42_ has been explored against the asexual stage of malaria parasite [3,19,20]. Presence of limited number of T-cell epitopes of MSP-1_19_ and thus compromised immunogenicity was overcome using the *Pv*MSP-1_42_, a homologue of *Pf*MSP-1_42_ in *P. vivax* and high serum antibodies against MSP-1_19_ were quantified [21].

Combination vaccine for malaria is likely to be more effective than vaccines based on a single antigen, and attempts have been made to develop a malaria vaccine using a mixture of more than one antigen or by combining immunologically relevant proteins of the target antigens as fusion proteins [22,23,24]. Therefore, constructed fusion chimera (MSP-Fu_24_) consisting of *Pf*MSP-1_19_ and *Pf*MSP-3_11_ to produce corresponding recombinant MSP-Fu_24_ protein in *Escherichia coli* cells. [25]. This engineered chimeric fusion protein was used to determine its immunogenic potential so as to explore it as potential asexual blood-stage malaria vaccine antigen.

Advancing interest in the development of nanoscale lipid-based delivery approaches for effective transcutaneous immunization led us to show transcutaneous delivery potential of elastic liposomes (ELs), and thus enhanced immunogenicity of *P. falciparum* surface antigen, MSP-1_19_, was shown [3]. Nanoscale drug carriers such as lipid (conventional and engineered liposomes) [3,26,27] and polymeric nanocarriers [28,29] have shown their potential in malaria prophylaxis [30] and vaccine development [3], and many others [31] have shown the value of lipid nanovesicles.

Therefore, we wanted to take advantage of delivery potential of deformable EL to effectively deliver the recombinant *Pf*MSP-Fu_24_ protein for assessing the humoral and CMI response to achieve immunity against *P. falciparum*. ELs were formulated and characterized with respect to vesicle shape, size, entrapment efficiency, and, stability [32] and delivered *Pf*MSP-Fu_24_ protein through IM and TD route of vaccination aimed at achieving sizeable immune response shown at both the transcript (IFN-γ, IL-4, and IL-10) and protein levels (IgG1, IgG3, IFN-γ, IL-4,and IL-10). Here, we report that EL-mediated topical delivery of *P. falciparum* antigen, *Pf*MSP-Fu_24_, achieved significantly higher and long-lasting humoral(isotypes, IgG 1, and IgG3) and CMI response (IFN-γ and IL-4).Collectively, our results suggest efficient TD delivery of *Pf*MSP-Fu_24_ protein via characterized EL(s) to elicit humoral and CMI responses to confer protection against asexual blood stage of *P. falciparum*.

## 2. Materials and Methods

### 2.1. Materials

*P. falciparum* asexual blood-stage surface antigen fusion protein (*Pf*MSP-Fu_24_) was received as gift from Prof. V.S. Chauhan, ICGEB, New Delhi, India. Soya phosphatidylcholine (SPC) and KolliphorP 188 were provided by Dr. Neeraj K. Garg, University Institute of Pharmaceutical Sciences, Panjab University, Chandigarh, India. Span 80 was received as a generous gift from Institute of Pharmacy, Nirma University, Ahmedabad, India. Methanol (Merck Life Sciences, Darmstadt, Germany SA8F680023), chloroform (Merck Life Sciences, DL7F672832), Sephadex R G-75 (Sigma Life Science, Darmstadt, Germany G75120), and all other basic requirements for the preparation of ELs were received from the local vendors unless otherwise specified. ELISA kits IgG1 (Cat No. 88-50410) and IgG3 (Cat No. 88-50440) were procured from Thermo Fisher Scientific, Waltham, USA. Mouse ELISA MAX Deluxe Set IFN-γ (Cat No. 430804) and IL-4 (Cat No. 431104)were procured from BioLegend, CA, USA. IL-10 ELISA (Cat No. M0046) Kit was a kind gift from Labex, India. All other chemicals and reagents were procured from local vendors.

### 2.2. Preparation and Physicochemical Characterization of Elastic Liposomes

ELs were prepared as described elsewhere [33] with slight modifications as mentioned in our previously published article [3]. Lipids were cast as a stack of the thin film from their organic solution using rotary evaporator (Divya Scientific and Chemicals, Ahmedabad (Gujarat), India). Briefly, SPC and Span 80 (86:14% (*w*/*w*)) were dissolved in chloroform: methanol (7:3) mixture [3]. Then, 20 mL of prepared system was transferred into 1000 mL round bottom flask for the evaporation of organic solvent through rotary evaporator at 200 rpm for 60 min at 60 °C until all the organic solvent was fully evaporated. Thin film of lipid was formed in a flask [33,34], which was suspended with phosphate buffer saline (PBS) containing *Pf*MSP-Fu_24_ (15 µg/mL) and rotated at room temperature (RT) for 3 h to ensure proper hydration of prepared thin lipid film. The prepared EL formulation was sonicated (Probe sonicator, Jay Chemical, Ahmedabad, India) to get unilamellar vesicles from the multilamellar vesicles [3,33,35] for 5 min at 110W (10s working, 5s off) to reduce the size and create homogeneity in the liposomal suspension, and sonicated liposomes were centrifuged at 5000 rpm for 10 min and the supernatant containing liposome was collected and stored at 4 °C for further experiments. Prepared elastic liposomal formulation was characterized with respect to size, polydispersity index, (Malvern Zeta Sizer, Institute of Life Science, Ahmedabad University, Gujarat, India), and zeta potential (Central University of Gujarat, Gandhinagar, Gujarat, India). EL formulations were analyzed through scanning electron microscope (SEM) to confirm external morphology (texture) and chemical composition. The unilamelarity and spherical shape of ELs was confirmed by transmission electron microscope (TEM) at central instrumentation facility at AIIMS, New Delhi, India.

### 2.3. Characterization of Merozoite Surface Chimeric Fusion Protein (PfMSP-Fu_24_)

The purity and integrity of *Pf*MSP-Fu_24_ was assessed by Coomassie Brilliant Blue (CBB) staining (Himedia, Mumbai, India RM344) *Pf*MSP-Fu_24_ separated on a reducing SDS-PAGE (sodium dodecyl sulfate polyacrylamide gel electrophoresis) (Himedia, Mumbai, India) gel. *Pf*MSP-Fu_24_ was extracted by dissolving the formulated elastic liposomes in 2mLTriton-X100 (CDH, New Delhi, India 9002-93-1). The extracted *Pf*MSP-Fu_24_ was concentrated by precipitation method using absolute ethanol as given in our previously published article [3] and loaded on 6% stacking gel and subjected to electrophoresis on a 12% separating gel at 100 V.

### 2.4. Separation and Purification of *Pf*MSP-Fu_24_

Separation and purification of *Pf*MSP-Fu_24_ from the formulated ELs was carried out as reported elsewhere [36]. In brief, Sephadex R G-75 (10% *w*/*v*) was swelled for 48 h in PBS and filled in 2 mL syringe having Whatman filter paper of same size to prevent the leakage of Sephadex beads from the syringe. Sephadex beads were loaded repeatedly onto 2 mL syringe, and PBS in excess was removed by centrifugation (2000 rpm for 3 min) until 2 mL column was reconstituted with Sephadex [36]. Purification of *Pf*MSP-Fu_24_ was carried out using the prepared minicolumn, and 400 µL *Pf*MSP-Fu_24_ sample was loaded on the column and centrifuged at 500× *g* (686 rpm) for 10 min and re-centrifuged at 1000× *g* (2988 rpm) for 3 min. Purified sample was collected in a centrifuge tube, and unentrapped antigen present inside the pores of beads was extracted by washing column with PBS and centrifuged at 1000× *g* (2988 rpm) for 3 min.

### 2.5. Estimation of Entrapment Efficiency

#### 2.5.1. Standard Calibration Curve of *Pf*MSP-Fu_24_

Stock solution of *Pf*MSP-Fu_24_ was prepared in PBS (pH 7.4) having a concentration of 20–60 µg/mL *Pf*MSP-Fu_24_. Then, 25 µL from each aliquot was transferred to a 2 mL micro-centrifuge tube and 25 µL PBS was kept as blank. Further, 1mL Bradford reagent (Himedia, Mumbai, India ML106) was added into each microcentrifuge tube, mixed well by vortexing and incubated at RT for 10 min, and absorbance was measured at 595 nm using UV–visible spectrophotometer.

#### 2.5.2. Determination of Entrapment Efficiency

The unentrapped *Pf*MSP-Fu_24_ elastic liposome formulation was separated using minicolumn centrifugation method to determine entrapment efficiency as reported in our published article [3]. For this, 1 mL purified suspension was transferred to a new microcentrifuge tube and mixed with 200 µL (0.1%) Triton X-100 [37] by vortexing for 1–2 min [38], and suspension was allowed to stand at RT for 10 min and mixed with 1mLchloroform and mixture was vortexed. Mixture was then centrifuged at 10,000 rpm for 10 min at 4 °C, and supernatant was collected. Further, 25 µL supernatant was transferred to a new Eppendorf tube having 1mL of Bradford reagent, and suspension was mixed well followed by the incubation for 10 min at RT. Absorbance was measured at 595 nm using UV–visible spectroscopy (Agilent Technologies Cary 60 Uv-Vis Spectrophotometer, Santa Clara, CA, USA), and entrapment efficiency was calculated as follows:

$$\%\;Entrapment\;Efficiency\;=\;\frac{Amount\;of\;PfMSP-Fu24\;in\;elastic\;liposome}{Total\;amount\;of\;PfMSP-Fu24\;added}\times 100$$

### 2.6. Immunogenicity Immunizations and Sample Collection

For this, 4–6-week-oldBALB/c mice were received from Zydus Research Center, Ahmedabad, India, to carry out immunization studies. Animal protocols were approved by the Institutional Animal Ethical Committee (IAEC) (IS/PHD/19/036), Institute of Pharmacy, Nirma University, Ahmedabad (Gujarat), India. These animals were provided with water *ad libitum* and maintained in pathogen-free conditions with convenient access to food and water. Grouping of animals was done in a random manner (Table 1). The hairs from abdomen of mice were removed using hair removal cream on the abdominal region, and then animals were allowed to rest for 24 h (for TD immunization). Prior to the immunization, skin was kept hydrated with normal-saline for the smooth delivery of antigen through TD route of vaccination. Further, in both the routes (IM and TD) boosting dose was given on day 14 with formulation(s) containing the same dose of *Pf*MSP-Fu_24_, and sera was collected on day 0 (baseline control) prior to immunization. Blood sampling was done on day 14, 21, 28, 42, 56, and 70, and serum was separated and stored at −80 °C until further use [3].

Mice in the experimental groups received EL-laden *Pf*MSP-Fu_24_ (EL-*Pf*MSP-Fu_24_) (11µg) and protein control groups received naked *Pf-*MSP-Fu_24_ (11µg). The vehicle control groups received normal saline and EL-entrapped PBS (EL-PBS).

### 2.7. Tissue Processing, Isolation of RNA for Gene Expression, and cDNA Synthesis

Group of animals receiving different *Pf*MSP-Fu_24_ formulations were euthanized on day 42and total RNA was extracted from spleen and lymph nodes. Animals from each group were euthanized to harvest spleen, lymph node, and kidneys to observe the morphology of organs other than lymph nodes and spleen and stored at −80 °C until further use. For that purpose, 8–15 mg of lymph node and spleen were homogenized to extract RNA as per manufacturer’s recommendations (GeneJET RNA Purification Kit, ThermoFisher Scientific, Catalogue No. K0731), and extracted RNA was stored at −80 °C until further use [39].

First Strand cDNA Synthesis Kit system (Thermo Scientific™ Revert Aid™ (Catalogue No.K1622) was used for the synthesis of cDNA. Thawed RNA was mixed with briefly centrifuged component of the kit, and reagents were added as per the manufacturer’s instructions. After completion of the synthesis, the product can be used for PCR (Catalog No. Z316091) (Eppendorf® Mastercycler, Eppendorf, Hamburg, Germany) applications directly or stored at −80 °C until further use.

### 2.8. RNA Quantification for Gene Expression Study

Mice from all groups (Table 1) were euthanized post immunization and RNA, secondary lymphoid organs (spleen and lymph node) were extracted and RNA was isolated using Thermo Scientific RNA Purification Kit, USA, and was quantified using the NanoDrop (737501) (Genova Nano Micro-volume Life Science Spectrophotometer, Jenway, Staffordshire, UK 737501) (Table 2).

#### 2.8.1. Reverse-Transcription Polymerase Chain Reaction (RT-PCR) Assay

Synthesized cDNA was further amplified using polymerase chain reaction (PCR) (Code No. RR310Q). Here, we used Emerald Amp GT PCR Master Mix having DNA polymerase, optimized reaction buffer, dNTPs, and density reagent. The premix also contains a vivid green dye that is used to separate blue and yellow dye fronts when run on an agarose gel. PCR was carried out for β-actin (house-keeping gene), IL-4, IL-10, and IFN-γ for 30 cycles using the PCR conditions given in Table 3.

The information on primer sequence used for said genes has been provided in Table 4.

#### 2.8.2. Quantitative PCR (qPCR)

We had carried out the gene expression analysis using real-time (RT)PCR as well as quantitative PCR (qPCR) quantify the up-/down regulation of genes (IL-12β, IFN-γ, TNF-α, and TGF-β)to unravel the role of the said genes responsible for conferring protection against *Pf*MSP-Fu_24_ delivered via elastic liposomes (EL-*Pf*MSP-Fu_24_) following transdermal and intramuscular route of administration. qPCR was performed on the ThermoScientific QuantStudio 3 qPCR system using SYBR^®^Premix EX Taq^TM^II (Clontech, TaKaRa Cat.=RR820A). The experiment was performed in duplicate and following PCR conditions were adopted: 94 °C 2min, followed by 40 cycles of 10 s at 94 °C, 10 s at 59 °C, and 34 s at 72 °C. The 2^−^^△△CT^ method was used to calculate fold regulations. The fold regulation of the gene in each group was calculated relative to its expression in the controlled samples for that gene and was calculated using GAPDH as the house-keeping control (Table S1).

### 2.9. Serum Estimation of Specific *Pf*MSP-Fu_24_ Antibody Levels

ELISA assays were carried out for IgG1 and IgG3. Two plates (provided with the kits) were coated with capture antibody diluted in sterile PBS and incubated overnight at 4 °C. Following 3 washes with wash buffer 0.05% Tween 20in PBS, plates were blocked with 250 µL blocking buffer for 1 h with 200 μL/well reagent diluent (R&D Systems, DY995). The serial dilutions of serum test samples, from untreated animals were prepared in reagent diluent and incubated with 50 μL/well overnight at RT. The plates were washed 4 times followed by addition of 100 μL HRP-labeled mouse IgG1and IgG3 diluted (1:100) in the reagent diluent to each and every well and incubated for 2 h. Plates were then washed 4 times followed by the incubation for 20 min with streptavidin-horseradish peroxidase (1:400) diluted in the reagent diluent, and 100 μL was added to all wells. Plates were washed 6 times and substrate was added and allowed to stand for 15 min for the development of color. The enzymatic color development reaction was terminated using stop solution (2N H_2_SO_4_), and plate was read at 450 nm with optical correction at 570 nm. The representative test curves for IgG2a and IgG1 with the absorbance values were compared to the total serum Ig levels obtained and were plotted as arbitrary units (AU).

### 2.10. Cytokine-ELISA Analysis of Serum Samples

The cytokine analysis is performed as per the manufacturer’s protocol. The lyophilized mouse IFN-γ standard was reconstituted in 0.2 mL of 1X Assay Diluent A to make it up to 70 ng/mL standard stock solution, and reconstituted standard was allowed to stand for 15–20 min and vortexed properly prior to use. The dilutions were made starting from 1000 pg/mL to 7.8125 ng/mL. Similarly, IL-4 was prepared in a concentration range of 125–2.0 pg/mL. Thereafter, wells of flat bottom microtiter 96 well plates were coated with 100 µL diluted capture antibody, and coated plate was incubated overnight at 37 °C followed by 4 times washing with PBS-Tween 20 (0.05%). The uncovered reactive sites in wells were blocked by incubating with 200 µL of blocking buffer. It is followed by 1h incubation in a plate shaker at 37 °C followed by washing with PBS-Tween 20 (0.05%) and further incubation with 100 µL of diluted (1:100) serum for 2 h in humidified chamber. Plates were washed again with PBS-Tween 20 (0.05%), and 100 µL of diluted detection antibody solution was added to each well and incubated for 1 h. Plates were washed 4–5 times, and 100 µL of diluted avidin-HRP antibody solution was added to each well. Plates were then reincubated for an additional 30 min in humid chamber and washed with PBS-Tween 20 (0.05%) for 5 times. TMB substrate solution was then added to each well and incubated in dark for 20 min and reaction was stopped by 2N H_2_SO_4_, and plates were read and optical density of the reaction product was measured with Multiscan ELISA plate reader (Bio-Rad, CA, USA) at 450 nm (OD450). The incubation of diluted serum in antigen-coated wells served as negative control. The protocols for IFN-γ and IL-4 estimation were followed as per the manufacturer’s recommendations. Similar experimental protocol was followed for quantifying IL-10, and manufacturer’s recommendations were strictly followed.

### 2.11. Statistical Analyses

Statistical analysis was performed using Prism 8 (8.4.2) software (GraphPad Software, San Diego, CA, USA). Two-way ANOVA followed by Tukey multiple comparison tests after Shapiro–Wilk normality test was used for statistical analysis of the groups. Ns = not significant, * *p* < 0.05, ** *p* < 0.01, *** *p* < 0.001, **** *p* < 0.0001.

## 3. Results

### 3.1. Formulation and Characterization of Nanoscale Lipid Vesicular System (Elastic Liposomes)

**Figure 1:**
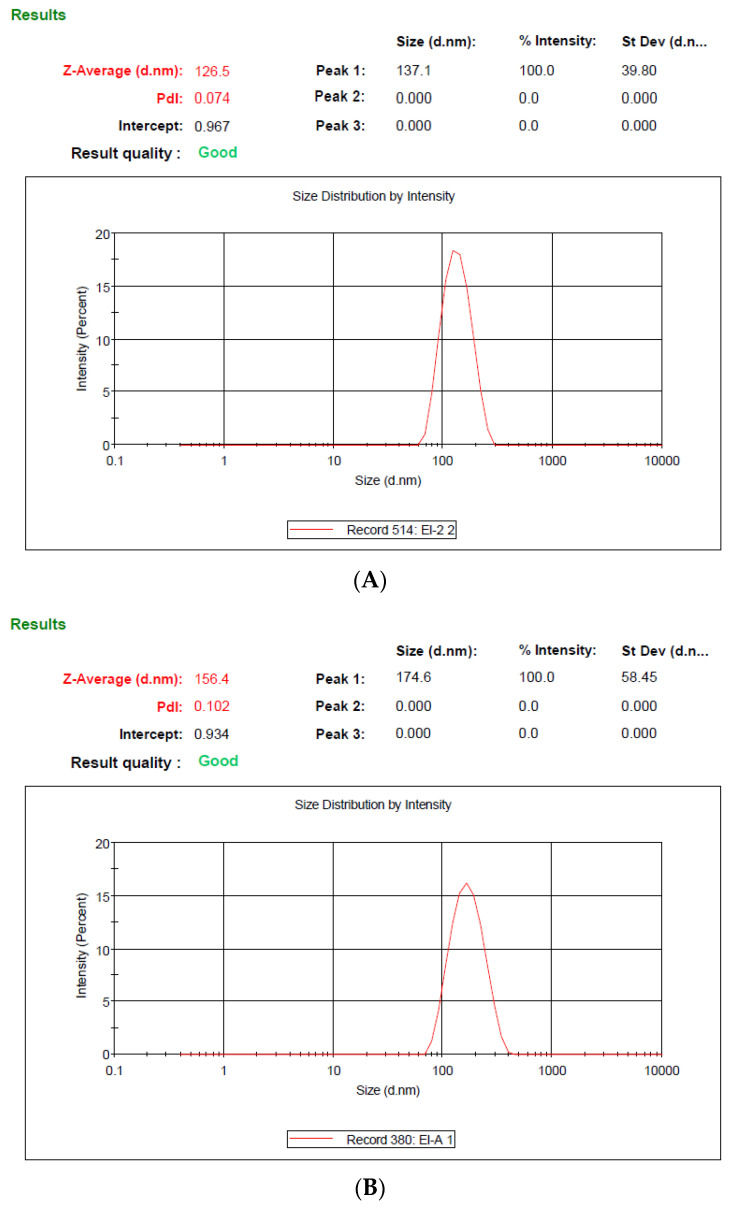

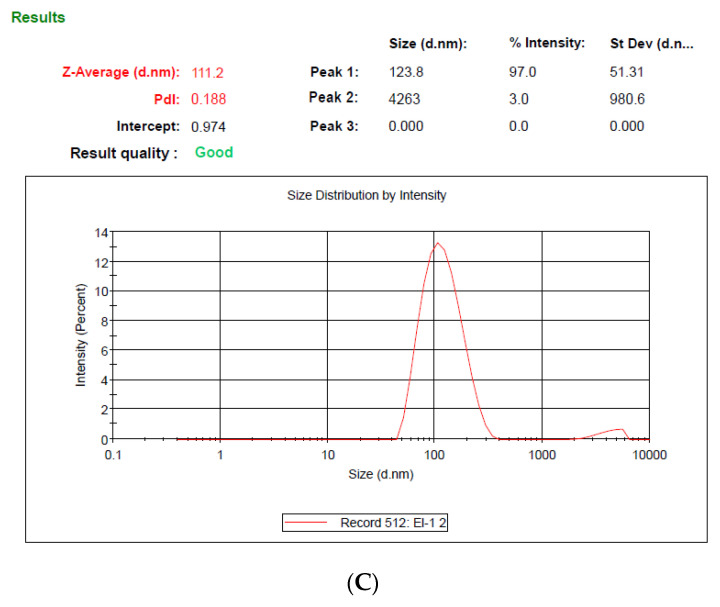
Particle size distribution of (**A**) phosphate buffer saline (PBS) reconstituted elastic liposomes, (**B**) bovine serum albumin (BSA)-loaded elastic liposomes (BSA-elastic liposomes (EL)), and (**C**) *Plasmodium falciparum* asexual blood-stage surface antigen fusion protein (*Pf*MSP-Fu_24_)-laden elastic liposomes (EL-*Pf*MSP-Fu_24_). Size was measured using zeta sizer (Malvern) and a single peak with 100% intensity at 137.1, 174.6, and 123.8 nm was seen for PBS, BSA, and *Pf*MSP-Fu_24_-loaded elastic liposomes, respectively. Polydispersity index was calculated to be 0.074, 0.102, and 0.188 for PBS, BSA, and *Pf*MSP-Fu_24_-loaded elastic liposomes formulations, respectively.

EL(s) were formulated by the conventional thin-film hydration method () and characterized with respect to morphology and texture analysis by SEM [33,40]. The model antigen (bovine serum albumin-BSA) was used to optimize the ratio of phosphatidylcholine (PC) to surfactant (S) following the method reported in our published article [3]. Consistent to earlier observations and findings [3,40], PC:S ratio (8.6:1.4) at which BSA-loaded elastic liposomes formulation was used to achieving better loading efficiency and elasticity, we used the same ratio to formulate *Pf*MSP-Fu_24_-laden elastic liposomes. BSA was used to formulate the elastic liposomes and characterized with respect to size, polydispersity index (PDI), and zeta potential (Figure 1).

**Figure 2:**
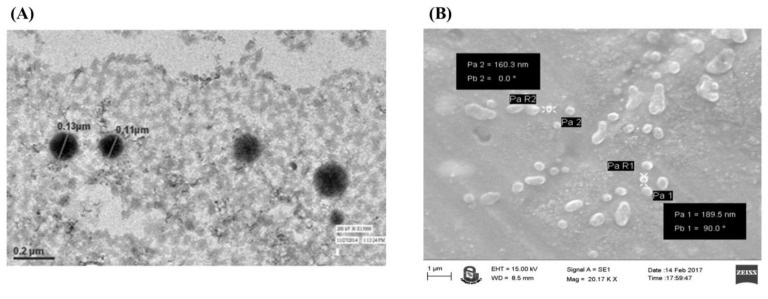
High-resolution, (**A**) TEM photomicrograph of EL-*Pf*MSP-Fu_24_ formulation at 100× magnification, and (**B**) SEM analysis of model antigen (BSA)-loaded elastic liposomes (EL-BSA) showing surface morphology of prepared formulation.

Size and PDI of EL formulations were measured by Malvern, zeta-sizer at the ILS, Ahmedabad. The mean diameters of PBS, BSA, and *Pf*MSP-Fu_24_-loaded ELs were estimated to be 137.1, 174.6, and 123.8 nm, respectively (Figure 1). Our results showed unilamelarity and spherical shape of elastic liposome loaded with *Pf*MSP-Fu_24_ (Figure 2A) confirmed by TEM and morphology of prepared elastic liposomal formulations was attested by SEM (Figure 2B).

In addition, results of zeta potential were further confirmed by dynamic light scattering (Table S2). These results were consistent to our previously published findings using *Pf*MSP-1_19_ secretory protein wherein mean diameter for PBS, BSA, and *P. falciparum* surface antigen, MSP-1_19_, were determined [3].

Collectively, our results suggest that all formulations fall within the acceptable size range (less than 200nm) and thus confer minimum nanoscale particles-induced cytotoxicity. PDIs of PBS, BSA, and EL-*Pf*MSP-Fu_24_ were estimated to be 0.074, 0.102, and 0.188, respectively. Since PDI is a measure of size-based heterogeneity of the sample, our results confirm the homogeneous size distribution and no agglomeration or aggregation ofEL-*Pf*MSP-Fu_24_ formulation suggesting the appropriate spherical dimension of liposomes (Table 5) (Figure 1 and Figure 2).

Zeta potential of the formulations falls in the range of 9–15 mV and hence showed stability of formulated lipid vesicles. The zeta potential of EL-*Pf*MSP-Fu_24_ was measured as 9.36 mV, which signifies its stability. Furthermore, surface charge of EL-*Pf*MSP-Fu_24_ was determined, which suggests the easy fusion of prepared elastic vesicles with the negatively charged membrane. This fusion leads to the release of the *Pf*MSP-Fu_24_ to the cytosol of target cells and maintained its structural integrity [41].

### 3.2. Surface Morphology Analysis by SEM

Surface morphology of formulated fusion protein-laden ELs was assessed through SEM showing uniform and spherical shape of lipid vesicles when loaded with antigen (Figure 2B and Figure S1). As earlier [3], EL formulation was prepared to achievethe sizeable and perdurable immune responses against transdermally administered surface antigen of human malaria parasite, *Pf*MSP-Fu_24_. These drug carriers were seen advantageous over other delivery systems due to their membrane flexibility and stress-endurance ability when surfactant (Span80) was used.

### 3.3. Entrapment Efficiency (EE)

Entrapment efficiency (EE) is the amount of recombinant protein (*Pf*MSP-Fu_24_) loaded onto the EL. We calculated the entrapment efficiency by determining the total quantity of protein loaded on to the ELs by plotting standard calibration curve (Table 6). The standard calibration curve for both BSA and *Pf*MSP-Fu_24_ was plotted using Bradford’s method for protein estimation (Figure S2).

### 3.4. Gene Expression Analysis by RT-PCR

**Figure 3:**
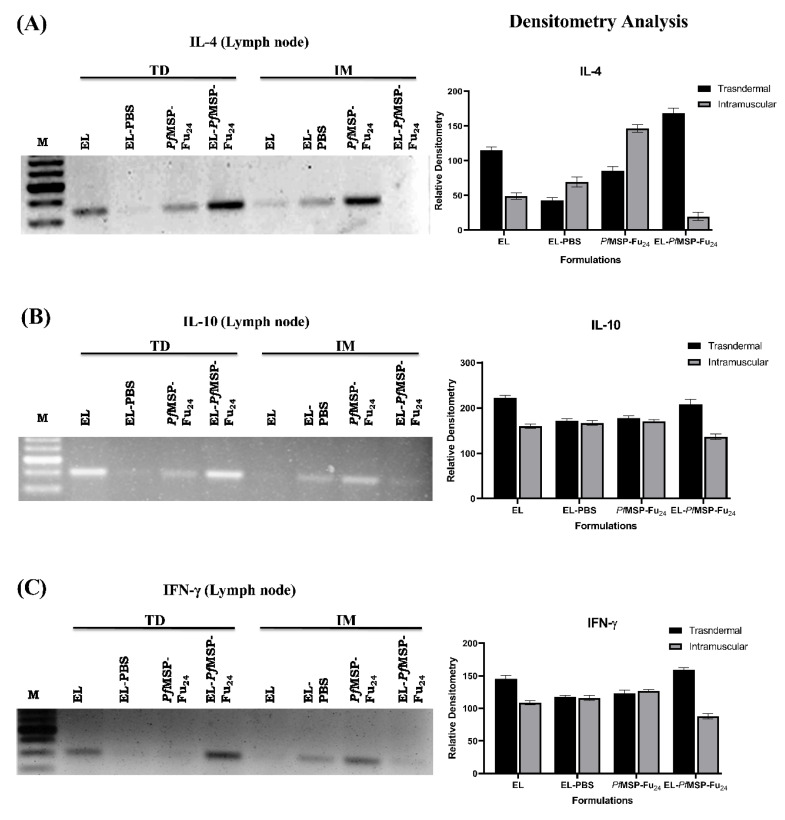
The expression analysis of (**A**) IL-4, (**B**) IL-10, and (**C**) IFN-γ by RT-PCR in the lymph nodes of BALB/c mice vaccinated with plain (*Pf*MSP-Fu_24_) and *Pf*MSP-Fu_24_-laden elastic liposomes (EL-PfMSP-Fu_24_).

**Figure 4:**
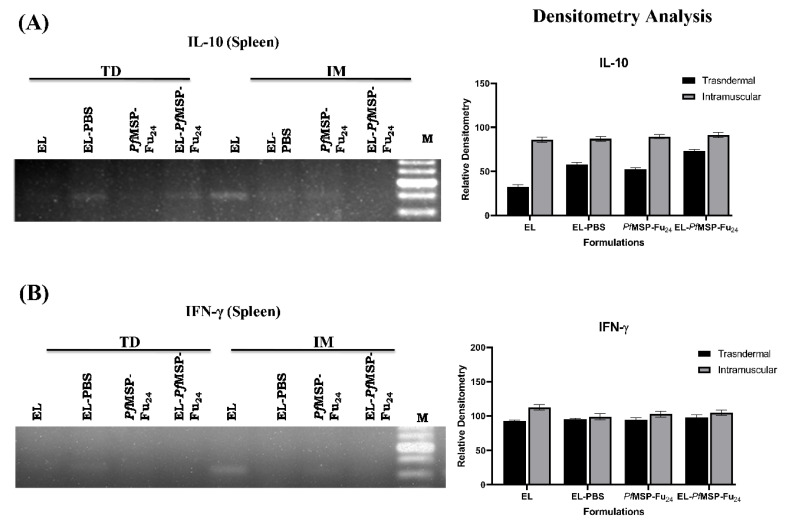
The transcript expression analysis of (**A**) IL-10 and (**B**) IFN-γ by RT-PCR in the spleen of BALB/c mice vaccinated with plain (*Pf*MSP-Fu_24_) and *Pf*MSP-Fu_24_-laden elastic liposomes (EL-PfMSP-Fu_24_).

Next, we decided to determine the gene expression of inflammation mediators by RT-PCR. cDNA was synthesized from extracted RNA and was amplified by PCR for the analysis of gene expression studies of β-actin, IFN-γ, IL-4, and IL-10. The obtained amplified PCR products were resolved on 1.5% agarose gel (Figure 3 and Figure 4), and resolved bands were used for analyzing the expression of different inflammatory cytokines in the deep-seated secondary lymphoid organs such as spleen and lymph nodes post immunization. The intensity of each band was normalized with the intensity of β-actin, the house-keeping control.

#### 3.4.1. Transcript-Level Expression of IL-4, IL-10, and IFN-γ in the Lymph Nodes and Spleen by RT-PCR

Greater transcript-level expression of IL-4 was determined in the lymph nodes of animals receiving *Pf*MSP-Fu_24_-laden ELs via transdermal route of vaccination than that seen with naked fusion protein (*Pf*MSP-Fu_24_) (Figure 3A). On the contrary, a higher expression of IL-4 in the lymph nodes of animals injected with naked fusion protein (*Pf*MSP-Fu_24_) was seen as compared to the intramuscularly administered *Pf*MSP-Fu_24_ (Figure 3A). The fusion protein-laden ELs when administered following transdermal route hardly saw a difference in IL-4 expression in spleen (data not shown). On the contrary, ELs-mediated intramuscular delivery of *Pf*MSP-Fu_24_ exhibited sizeable expression of IL-4 in the spleen as compared to that seen with the naked protein. However, it could not reach the statistical significance (data not shown).

We had conducted experiments to assess the levels of the immunoregulatory cytokine, IL-10, in the lymph nodes and spleen. There was scarcely any difference seen in the expression of IL-10 in lymph nodes of mice intramuscularly immunized with naked fusion protein (*Pf*MSP-Fu_24_) (Figure 3B). However, there was a sizeable difference in the lymph node levels of IL-10 expression seen with TD immunization of EL-mediated delivery of *Pf*MSP-Fu_24_ (Figure 3B). However, intramuscularly administered naked fusion protein and EL-mediated delivery of *Pf*MSP-Fu_24_ mounted significant splenic expression of IL-10 as compared to the transcutaneously applied naked or *Pf*MSP-Fu_24_-laden elastic liposome formulations (Figure 4A).

Since CMI plays a crucial role in the clearance of asexual blood-stage infection of *P. falciparum,* we decided to assess the changes in the transcripts of IFN-γ in the lymph nodes (Figure 3C) and spleen (Figure 4B). Our results show higher expression of IFN-γ in the lymph nodes of mice receiving *Pf*MSP-Fu_24_ through TD route of vaccination by the deformable nanoscale ELs and compared to the responses mounted by the intramuscularly administered ELs guided delivery of *Pf*MSP-Fu_24_ formulations (Figure 3C). Interestingly, we could not quantify the significant difference in the expression of IFN-γ in the spleen mounted in response to the naked delivery of *Pf*MSP-Fu_24_ or via ELs following the IM or TD route of vaccination (Figure 4B).

#### 3.4.2. Assessment of Transcript-Level Fold Regulation of IL-12β, IFN-γ, TNF-α, and TGF-β in Lymph Nodes by qPCR

**Figure 5:**
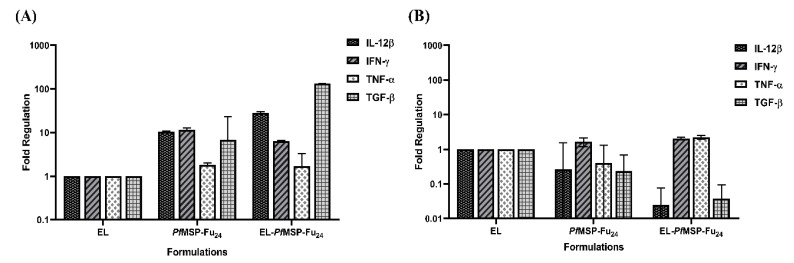
The assessment of transcript-level fold regulation of IL-12β, IFN-γ, TNF-α, and TGF-β genes from lymph nodes of BALB/c mice receiving plain (*Pf*MSP-Fu_24_) and elastic liposomes-laden (EL-PfMSP-Fu_24_) formulations via (**A**) transdermal and (**B**) intramuscular route by q-PCR. The fold change (in gene expression) value greater than one indicates a positive value or an upregulation of gene expression and the value less than one indicates a negative value or downregulation of gene expression.

We carried out the qPCR analysis of IL-12β, IFN-γ, TNF-α, and TGF-β (transforming growth factor beta) in the secondary lymphoid organ (lymph nodes) in both the groups receiving plain and elastic liposomes-laden *Pf*MSP-Fu_24_ through transdermal (Figure 5A) and intramuscular route of administration (Figure 5B). Our results showed the significantly higher transcript fold regulation in immunoregulatory molecule (TGF-β) with elastic liposomes-mediated transdermal delivery of *Pf*MSP-Fu_24_ (Figure 5A) as compared to the regulation seen with the elastic liposomes-mediated intramuscular delivery of *Pf*MSP-Fu_24_ (Figure 5B). Further, there was hardly a difference seen in the levels of TNF-α in the mice receiving the antigen through transdermal route (Figure 5A). On the contrary, we saw an increased fold regulation of TNF-α with EL-*Pf*MSP-Fu_24_ injected through intramuscular route (Figure 5B).

### 3.5. Assessment of In-Process Stability of Chimeric Fusion Protein, *Pf*MSP-Fu_24_

**Figure 6:**
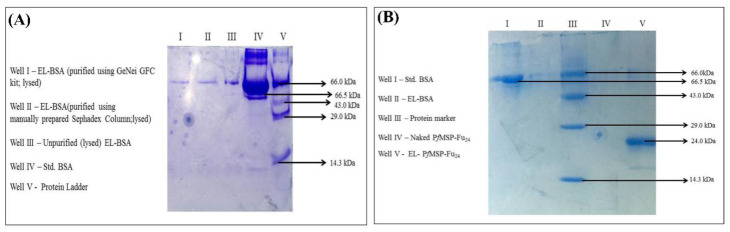
Assessment of in-process stability of different formulations by SDS-PAGE analysis for different formulation of plain and elastic liposomes loaded with (**A**) BSA and (**B**) EL-*Pf*MSP-Fu_24_. The bands were seen intact at 67 kDa (BSA) and 24 kDa (*Pf*MSP-Fu_24_).

The integrity of BSA and asexual blood-stage fusion protein of *P. falciparum* when loaded onto the elastic liposomes (EL-BSA and EL-*Pf*MSP-Fu_24_) was analyzed by SDS-PAGE. The clear visible bands for naked and protein extracted from ELs were observed on SDS-PAGE (12%) for BSA and *Pf*MSP-Fu_24_ at 67 and 24 kDa positions, respectively, against the standard molecular weight marker (Figure 6). Our results indicate the intact integrity of the fusion protein as no bands for the degradation product were seen on SDS-PAGE, and banding pattern of encapsulated antigen was similar to the native protein (Figure 6). Therefore, we did not see the procedural effect on the aggregation and cleavage of the fusion protein and thus suggests the intact integrity of the recombinant fusion protein during the encapsulation of *Pf*MSP-Fu_24_ in EL.

### 3.6. Role of Antibody Isotypes in Malaria Protection

**Figure 7:**
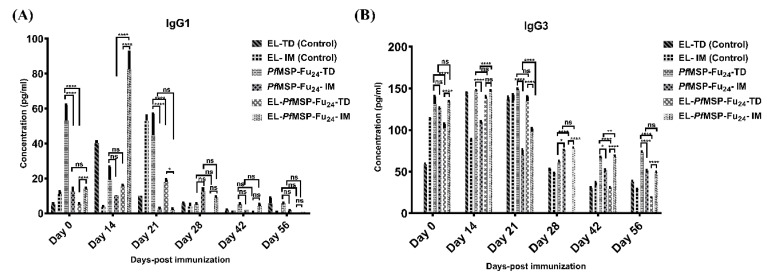
The specific IgG antibody response in mice vaccinated with plain *Pf*MSP-Fu_24_ and *Pf*MSP-Fu_24_-loaded elastic liposomal formulations. The statistical data are expressed as mean ± SE (n = 3 or 5). (* *p <* 0.05, ** *p <* 0.01, and *** *p* < 0.001). (**A**) IgG1 and (**B**) IgG3 isotype subclass of specific antibody (IgG) levels in mice immunized with *Pf*MSP-Fu_24_ antigen formulations. Mean ± SE (n = 6). (* *p* < 0.05, ** *p* < 0.01, *** *p* < 0.001 and **** *p* < 0.0001).

We next decided to confirm the contribution of subclass of immunoglobulins (IgG1 and IgG3) for conferring protection against asexual blood-stage infection of *P. falciparum*. We determined the levels of IgG1 and IgG3 in the serum samples collected from mice receiving experimental formulations; naked (*Pf*MSP-Fu_24_) and EL-*Pf*MSP-Fu_24_ through TD and IM routes. Free/empty EL were injected through transdermal (EL-TD) and intramuscular routes (EL-IM) and served as negative control in the experiment (Figure 7A,B). The difference in IgG1 response were seen significant (*p <* 0.001) withEL-*Pf*MSP-Fu_24_ administered through TD route compared to those seen with the IM administered EL-*Pf*MSP-Fu_24_ formulation mainly on day 21 (7 days post boosting on day 14). On the contrary, transdermally administered naked *Pf*MSP-Fu_24_ showed higher IgG1 responses than those seen with intramuscularly injected protein on day 0 (Figure 7A). Interestingly, a steep increase in IgG1response was seen with intramuscularly administered and EL-mediated IM delivery of *Pf*MSP-Fu_24_ formulation on day 14 (Figure 7A). IgG1 responses were seen to diminish day 21 onwards until day 56. IgG3 levels were seen significantly higher (*p* < 0.001) at almost all time points except on day 21 post boosting with intramuscularly administered EL-*Pf*MSP-Fu_24_ compared to the transdermally injected EL-*Pf*MSP-Fu_24_ (Figure 7B).

The distribution of IgG1 and IgG3 subclass of antibody isotypes was found greater with EL-*Pf*MSP-Fu_24_ than intramuscularly injected *Pf*MSP-Fu_24_ and negative control. Our results suggest better IgG3 response with intramuscularly administered *Pf*MSP-Fu_24_ upon priming on day 0 and 7 and boosting on day 14. However, we observed significantly higher response elicited by the TD immunization of fusion protein formulation when delivered via transdermal route compared to the intramuscular route on day 21 following boosting on day 14 (Figure 7B). Naked fusion protein (PfMSP-Fu_24_) exhibited significant (*p <* 0.001) IgG3 titer upon injection through TD immunization as compared to the IM route of immunization on day 0, 7, 14, 21, 42, and 56 (Figure 7B).

### 3.7. Cell-Mediated Immune Response Elicited against Asexual Blood-Stage Infection of P. falciparum

**Figure 8:**
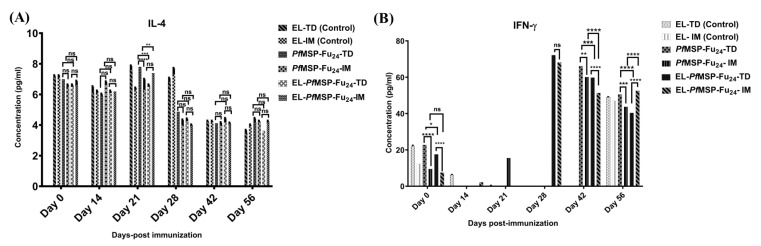
Assessment of the cell-mediated immune (CMI) responses (**A**) IL-4 and (**B**) IFN-γ against asexual blood-stage infection of *P. falciparum* from mice vaccinated with plain *Pf*MSP-Fu_24_ and *Pf*MSP-Fu_24_-loaded elastic liposomal formulations. The statistical data are expressed as mean ± SE (n = 3 or 5). (* *p* < 0.05, ** *p* < 0.01, *** *p* < 0.001 and **** *p* < 0.0001).

Both cellular and humoral arms of the adaptive immune system play an instrumental role in the clearance of *Plasmodium* from the body. These immune responses are critically dependent on α/β_CD4_ lymphocytes and established that T cells play a crucial role in the clearance of asexual blood-stage malaria parasites by activating the secretion of cytokines and that CD4 T cells constitute two functionally different subsets, i.e., Th1 (IFN-γ producing) and Th2 (interleukin-4 [IL-4]/IL-5-producing) cells. IL-12, IFN-γ, and TNF-α are responsible for provoking immunity and thus conferring protection against asexual blood-stage malaria infection [42]. As reported elsewhere [43] and confirmed by our group [3], IL-4 exerts the inhibitory effect on the IFN-γ production when induced by IL-12 priming. Therefore, we quantify the expression of IL-4 (Figure 8A), IFN-γ (Figure 8B), and IL-10 (Figure S3) in the serum samples collected from mice immunized with *Pf*MSP-Fu_24_ chimeric protein formulations (empty andEL-*Pf*MSP-Fu_24_) through TD and IM routes of vaccination. Our results suggest that plain and *Pf*MSP-Fu_24_-loaded EL fusion protein did not stimulate Th2 cells and thus secretion of IL-4 (Figure 8A) did not reach the threshold to inhibit the production of IFN-γ. IFN-γ, a cell-mediated immune effecter, plays an important role in conferring resistance to blood-stage malaria infection [44]. Moreover, the activation of monocyte-derived human macrophages by IFN-γ resulted in the induction of phagocytosis leading to the killing of malaria parasites [3]. We saw mixed results of IFN-γ, and transdermally injected EL-*Pf*MSP-Fu_24_ exhibited significant IFN-γ production compared to the response seen in the intramuscularly immunized mice (Figure 8B); significant levels were maintained until day 42 post immunization, and then, dampening effect of IFN-γ levels was observed (Figure 8B).

The balance between pro- and anti-inflammatory immune responses of the host plays a critical role in determining the outcome of malaria pathogenesis. Interleukin-10 (IL-10) is believed to play a critical role in the regulation of host immune response as well as switching from Th1 to Th2 responses [45,46]. In addition, IL-10 orchestrates the reversal of Th1/Th2 immune dominance during infectious disease pathogenesis and progression, and earlyIL-10 production has been associated with host’s susceptibility towards infection [47,48]. Therefore, we decided to estimate the serum secretion of IL-10 in the mice vaccinated with empty and *Pf*MSP-Fu_24_-laden ELs through IM and TD routes (Figure S3). We did not see the secretion of IL-10 on day 0, 7, and 14 post immunization. However, on day 21 and 42 post immunization, there was a sizeable secretion of IL-10 seen in the serum of animals receiving EL-*Pf*MSP-Fu_24_ and free *Pf*MSP-Fu_24_ as compared to the intramuscularly injected *Pf*MSP-Fu_24_ (Figure S3). Our results consistent to others findings show that this anti-inflammatory cytokine during late stages of infection plays a very crucial role in controlling inflammatory responses and thus preventing tissue damage [46].

## 4. Discussion

**Scheme 1:**
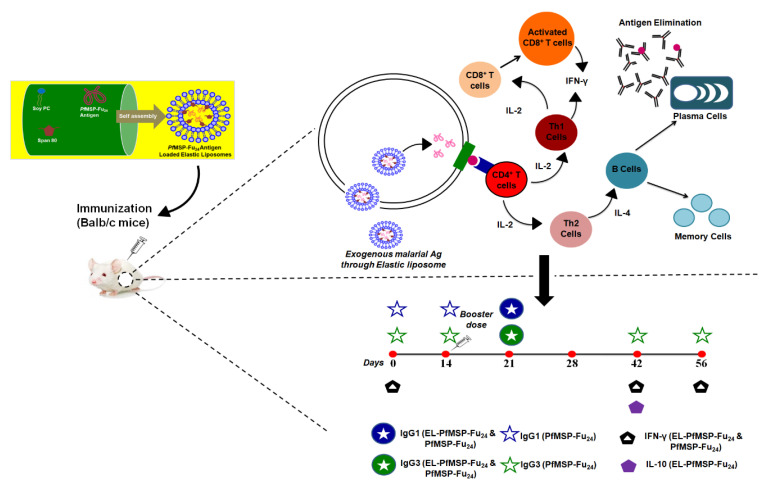
Elastic liposomes-laden *Plasmodium falciparum* asexual blood-stage surface antigen fusion protein (*Pf*MSP-Fu_24_) formulation showing the induction of protective and regulatory antigen-specific immune response against *P. falciparum.*

Vaccination provides one of the most cost-effective preventive measures against illness and deaths resulting from the systemic inflammatory disorders. Skin has been an attractive site for the effective expression of antigen and eliciting antibody-mediated humoral immune responses [33]. Noninvasive immunization through topical route has been used to express, and immune responses elicited by plain antigen and delivery systems-laden antigens were compared. However, inefficient penetration through intact skin has been one of the major impediments [49,50] towards mounting sizeable and perdurable immune responses when vaccinated through TD route. Our previously published finding confirmed the role of these deformable ELs for the efficient transdermal delivery of carboxyl-terminal 19 kDa fragment of merozoite surfaceprotein-1, *Pf*MSP-1_19_, and showed better penetration through the intact skin to LCs (Langerhans cells) and other abundantly populated APCs. We saw induced humoral and cell-mediated immune responses for eliciting protective immune response against *P. falciparum* [3].Therefore, subcutaneous administration has shown its potential and emerged as viable route of vaccination for antigen formulations to achieving enhanced immunogenicity. IM route of administration, however, has been the most commonly used route for the licensed vaccines, and multiple clinical trials outcome hardly showed any difference in the induced adaptive immune responses when antigens were delivered and compared for TD and IM routes [51]. Therefore, present study aimed at showing the relevance of these nanoscale, deformable lipid vesicles for the effective transdermal delivery of a novel chimeric fusion protein (*Pf*MSP-Fu_24_) for generating the vaccine-specific immune responses following TD and IM administration (Scheme 1).

We had optimized the phosphatidylcholine to surfactant ratio (8.6:1.4) [3] and therefore used this ratio to formulate ELs with acceptable particle size range and unilamellar spherical shape (Figure 1 and Figure 2, Figure S1 and Table 5). Our study shows better entrapment efficiency (Table 6) of *Pf*MSP-Fu_24_.However, greater entrapment efficiency of EL-*Pf*MSP-Fu_24_ than the model antigen BSA was attributed to the smaller size of fusion protein (24 kDa) than BSA (65 kDa).

The deformability and sensitivity of these ELs to water gradient across the skin layers prove this delivery carrier as a potential nanoscale vaccine delivery system for topical immunization in order to achieve the long-lasting and heightened immune response. Lipid vesicles reportedly follow the pilosebaceous route to bypass the stratum corneum barrier for the smooth entry of biomolecules including interferon, monoclonal antibodies, and DNA vaccines [52,53]. We believe ELs contributed to boosting the immune response as an “adjuvant” and therefore proved useful during TD vaccination.

Considerable immune responses against *Pf*MSP-Fu_24_ antigen with human compatible adjuvants were estimated by the skin-associated macrophages and dendritic cells, and a few studies were carried out by Mazumdar et al. [25,54], we decided to further explore the immunogenic potential of this novel chimeric fusion protein in BALB/c mice sans adjuvant. Therefore, in vivo assays were conducted to confirm the immnunoadjuvant effect of elastic liposomes and its potential to deliver *Pf*MSP-Fu_24_ for eliciting sizeable humoral and CMI immune response against asexual blood-stage infection of *P. falciparum* at molecular levels. Since cytokine IL-4 is known to play an important role in invoking type 2 immune responses and conferring immunity to parasites, early investigations using the in vitro culture systems suggested that the dominant role of IL-4 holds crucial in driving and shaping the CD4 Th2 subset differentiation toward expression of canonical type 2 cytokines IL-4, IL-5, and IL-13 [55,56]. Therefore, we sought to clarify the role of IL-4 expression and accumulation following a primary response to the *Pf*MSP-Fu_24_ antigen.We detected the greater expression of IL-4 secreted by CD4 T cells that appear in the draining lymph node and subsequent type 2 inflammatory response following transdermal immunization of *Pf*MSP-Fu_24_ delivered via ELs as compared to the immune responses provoked by the intramuscular delivery of *Pf*MSP-Fu_24_ mediated by EL. Since IL-4 is known to regulate the CMI activating factor (IFN-γ) by leaving an inhibitory effect on IL-12 enhanced priming for IFN-γ production, our results showed the higher mRNA expression in the early phase of immune activation when ELs mediates the transdermal delivery of *Pf*MSP-Fu_24_. On the other hand, IM delivery of naked *Pf*MSP-Fu_24_ exhibited higher mRNA expression of IL-4 (Figure 3A),and thus our findings are consistent to the earlier findings wherein early phase immune responses were reported better as compared to later stage of antigenic exposure [57].

IL-10 plays a critical role in the immunoregulatory networks and protects tissue from infection-induced inflammation during malaria pathology. There has been convincing mechanistic evidence from preclinical malaria models and patient data showing key roles for IL-10 in preventing several severe manifestations of malaria including development of anemia and damage to organs. Since IL-10 is like a double-edged sword as it may both suppress important antiparasitic immune responses and affect the functions of Th1 cell responses. It is, however, known to protect the host from tissue damage [58]. Considering the fact that IL-10 may promote antiparasitic antibody production by B cells and may be detrimental for malaria pathogenesis and progression, our results of RNA expression of IL-10 in secondary lymphoid organs (lymph nodes and spleen) suggest the better expression of IL-10 in spleen with intramuscularly injected EL-*Pf*MSP-Fu_24_ compared to the TD immunization of antigen. We think, if IL-10 production could be modulated by specific cell populations or subsets to protect tissue from inflammatory mediators, the underlying mechanisms and clinical benefits may be achieved. However, it needs further investigations to identify the unique cellular signaling pathways operated for the regulation of IL-10 production in different cell populations.

As IFN-γ plays an important role in the clearance of asexual blood-stage infection of *P. falciparum,* our results at transcript-level expression of exhibiting sizeable expression of this CMI effecter with transdermal immunization of *Pf*MSP-Fu_24_ in the draining lymph nodes advocating for conferring protection against blood-stage infection of *P. falciparum.* Our results, as others [59], are in agreement to previously published findings [3,60] wherein we have shown the protection conferred by the *Pf*-MSP-1_19_ against *P. falciparum.*

We had shown the qualitative fluorescence emitting from white pulp of spleen when FITC-labelled-BSA was delivered to via elastic liposomes through lymphatic or systemic circulation. Moreover, the histopathology conducted on the skin sections of control and treated animals confirmed the efficient TD delivery of *Pf*MSP-1_19_ through elastic liposomes. The in-process stability is crucial for provoking the antigen-specific immune response as the entrapment (of antigen) procedure might denature/deactivate and the antigenic protein may not evoke significant and perdurable immune response. Our results showed the intact integrity of *Pf*MSP-Fu_24_; conditions of preparation, which did not cause any cleavage or irreversible aggregation of protein; and unaltered immuno-reactivity of antigen-loaded elastic liposomes (Figure 6).

Further, our results showed the sustained (8 weeks) and comparable serum antibody isotypes (IgG1 and IgG3) responses against transdermally administered EL-*Pf*MSP-Fu_24_ and compared with naked antigen and intramuscularly injected liposomal formulations in BALB/c mice. These lipid vesicles have shown their delivery potential enabling the sustained and heightened antigen-specific immunity [3,21,61]. We have shown in our earlier finding that protective immunity in BALB/c mice is correlated with the level of antibodies seen in the serum against *P. falciparum* and thus conferred protection is antibody isotype dependent. Our findings are in conformity with the study showing increased levels of *P. falciparum*-specific cytophilic antibodies (IgG1 and IgG3) levels in the individuals living in endemic areas [62,63,64]. IgG1 and IgG3 confers protection against clinical malaria [65,66] by the neutralization of parasites through the direct inhibition or by opsonization [67,68]. Therefore, our results with sizeable IgG1 and IgG3 responses quantified against *Pf*MSP-Fu_24_ when delivered through transdermal route as compared to responses seen with intramuscularly administered EL-*Pf*MSP-Fu_24_ postbooster dose (Figure 7A,B) are of particular importance.

Our data are consistent to the previously reported findings suggesting that particulate antigens can be processed and presented either by MHC class I or II by the dendritic cells and macrophages, which stimulatesTh1 and Th2 lymphocyte subpopulations, whereas soluble antigens are exclusively presented by class II MHC, stimulating the Th2 response [69]. Based on our results, we believe that the elastic liposomal formulations may elicit both Th1 and Th2 immune response. APCs having both MHC-I and MHC-II molecules leading to the processing and presentation of antigen through endocytic and cytosolic pathway and thus evoke the humoral and cellular immune response. As there is suboptimal uptake of plain *Pf*MSP-Fu_24_ by immunologically active cells, we think that elastic liposomes-mediated effective and sustained delivery of *Pf*MSP-Fu_24_ may be attributed to the immunological responses. Thus, the structural versatility of ELs, their ability to encapsulate variety of antigens irrespective of size and solubility, favorablebio-distribution, and intracellular targeting profile make these lipid vesicles an effective means for the immuno-potentiating and effective delivery of vaccine candidate even with any adjuvant.

As T-cell-mediated immunity plays a crucial role to clear blood-stage parasite malaria parasite and IL-4-controlled CMI activating factor (IFN-γ), we determined IL-4 and IFN-γ responses against EL-*Pf*MSP-Fu_24_ and plain *Pf*MSP-Fu_24_ when delivered through topical and intramuscular route. We hardly observed any marked difference in the serum levels of IL-4 with *Pf*MSP-Fu_24_-loaded elastic liposomal formulations administered transdermally or intramuscularly (Figure 8A). In conformity with the published findings, IFN-γ levels were seen significantly higher with the transdermally delivered and compared with the responses generated by the intramuscular vaccination fusion protein. The induced cell-mediated immunity against asexual blood-stage infection of *P. falciparum* and protection was conferred.

Role for conferring protection against malaria infection has been validated using IFN-γ or its receptor gene KO mice. The higher rate of mortality and morbidity in IFN-γ knock-out mice was observed upon challenged with *P. Chabaudi.* These findings gave valuable insights to the crucial role played by this CMI effecter to control parasitemia and survival of the host during primary infection [70,71,72]. Recent studies, as earlier [73,74], has confirmed the requirement of IFN-γ responses to several pre-erythrocytic and blood-stage *P. falciparum* vaccine candidates as decrease was seen in the absence of malaria transmission. Therefore, human malaria infection is associated with protection rendered by IFN-γ [75]. Present study demonstrates that suppressed levels of protein level of endogenous IL4 allowed the higher production of IFN-*γ*. ELs mediate the effective delivery of *Pf*MSP-Fu_24_ and elicit significantly higher cell-mediated immune response, and our data suggest the critical role of endogenous IFN-*γ* in regulating protective immune responses against asexual blood-stage *P. falciparum* infection. Furthermore, our findings are in conformity with others and surface the role of IFN-*γ* in rendering protection to rodent as well as human parasite. The higher mortality and morbidity due to malaria infection [70,74] suggesting a role for this CM immune effecter towards conferring protection. We achieved sizeable serum levels of IFN-γ in the mice receiving ELs-loaded malaria antigen compared to the other formulation, and our findings confirm the important role played by IFN-γ in conferring resistance to blood-stage malaria infection.

In the end, we wanted to confirm whether IL-10 maintain the delicate balance between pro- and anti-inflammatory immune responses of host and malaria parasite and modulates the host’s immune system to produce local/circulatory regulatory T cells (Tregs). Tregs are involved in the immunosuppressive activity [58,76], and expression of IL-10 in the later stage of antigenic exposure with the elastic liposomes-mediated transdermal delivery of *Pf*MSP-Fu_24_ as compared to the IM route confirmed its role in maintaining the balance between pro-and anti-inflammatory responses during malaria infection. The greater expression of TGF-β in the lymph nodes quantified (by qPCR) in the transdermally injected animals advocates for the immunoregulatory control of our subcutaneous formulation along with the protein level expression of IL-10.

## 5. Conclusions

Drug resistance and unavailability of an effective vaccine prompted us to assess the immunogenic potential of newly engineered chimeric fusion protein (*Pf*MSP-Fu_24_). Our findings showed that ultradeformable nanoscale elastic liposomes may overcome the skin permeability barrier and deliver antigenic payload to the immunologically active cells of the skin and draining lymph nodes. Elastic liposomes through their enhanced elasticity [3], better antigen presentation, controlled antigen release, immuno-adjuvant properties, and greater entrapment efficiency could be utilized as an effective delivery platform to deliver *P. falciparum* vaccine candidates for inducing the antimalarial immunity. The noninvasive administration of elastic liposomes offers needle-free vaccine delivery and higher antibody titer along with sizeable cell-mediated immune response to clear asexual blood-stage infections of *P. falciparum.* Further studies using elastic liposomes to deliver the novel antigens shall contribute to the vaccine development efforts against malaria. In the end, present study is an attempt to explore *Pf*MSP-Fu_24_, a chimeric protein, as potent asexual blood-stage vaccine candidate and its immunogenic potential.

## Figures and Tables

**Table 1:** Grouping of animals for immunization protocol.

Group 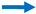	Vehicle Control	Protein Control	Experimental Control	Immunization		Serum Collection (Day)
Route 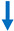				Priming (Day)	Boosting (Day)	
Intramuscular (IM)	4	3	5	1	14	0, 14,21,28,42,56, and 70
Transdermal (TD)	4	3	5	1	14	0, 14, 21, 28, 42, 56, and 70

**Table 2:** RNA extracted from homogenized spleen and lymph nodes.

Sr. No.	Formulation	Spleen		Lymph Node	
		Concentration (ng/µL)	Λmax 260/280 nm	Concentration (ng/µL)	Λmax 260/280 nm
1	EL-TD	72.485	2.00	318.640	2.00
2	EL-IM	117.790	2.00	281.410	2.00
3	*Pf*MSP-Fu_24_-TD	155.740	2.09	236.980	2.00
4	*Pf*MSP-Fu_24_-IM	94.822	1.40	209.810	2.00
5	EL-*Pf*MSP-Fu_24_-TD	74.063	1.85	283.870	2.00
6	EL-*Pf*MSP-Fu_24_-IM	231.190	2.00	69.576	1.90

**Table 3:** PCR condition for different gene.

PCR Steps	Temperature (°C)	Time (s)
1. Initial denaturation	95	180
2. Denaturation	95	30
3. Annealing *	53–59	35
4. Extension	72	45
5. Final extension	72	600

* Depends upon the respective primers of gene. Step 2 to 4: repeat 30 cycle.

**Table 4:** Primer sequence used for RT-PCR.

Cytokine Gene	Primer Sequences
β-actin	F: 5-GGAATCCTGTGGCATCCATGAAAC-3 R: 5-TAAAACGCAGCTCAGTAACAGTCCG-3
IL-4	F: 5-CGAATGTACCAGGAGCCATATC-3 R: 5-CCAGGAAGTCTTTCAGTGATGT-3
IL-10	F: 5-CTATGCTGCCTGCTCTTACTG-3 R: 5-CTCCACTGCCTTGCTCTTATT-3
IFN-γ	F: 5-CACACTGCATCTTGGCTTTG-3 R: 5-GCTGTTGCTGAAGAAGGTAGTA-3

F indicates “Forward primer” and R indicates “Reverse primer”.

**Table 5:** Determination of size and polydispersity index (PDI) of elastic liposomes.

No.	Formulation	Size (d.nm)	PDI
1	EL-PBS	137.1± 39.80	0.074
2	EL-BSA	174.6± 58.45	0.102
3	EL-*Pf*MSP-Fu_24_	123.8± 51.31	0.188

**Table 6:** The entrapment efficiency of bovine serum albumin (BSA) and *Plasmodium falciparum* asexual blood-stage surface antigen fusion protein (*Pf*MSP-Fu_24_)-loaded elastic liposomes.

Formulation Code	Quantity of Content after Rupturing of Elastic Liposomes (µL) A	Total Quantity of Entrapped in Elastic Liposomes (µL) B	% Entrapment Efficiency (EE) A/B∗100
EL-BSA	8.6	15	57.33%
EL-*Pf*MSP-Fu_24_	11	15	73.33%

## Data Availability

All figures and tables has been given in the manuscript and no other data is left to be disclosed.

## Supplementary Materials

The following are available online at https://www.mdpi.com/2079-4991/11/2/406/s1, Figure S1: (**A**–**C**) Thin lipid film formation 86:14 *w*/*w* soya lecithin liquid:Span-80 7:3 (*v*/*v*) chloroform: methanol (**D**) thin-film hydration suspension of lipid film in PBS (pH 6.5) 15 μg/mL BSA dissolved in 1× PBS (pH 6.5); Figure S2: Standard calibration curve drawn for (**A**) BSA and (**B**) *P. falciparum* asexual blood-stage surface antigen fusion protein (*Pf*MSP-Fu_24_) at 595 nm wavelength; Figure S3: Serum quantification of immunoregulatory cytokine, IL-10 in the mice vaccinated with plain *Pf*MSP-Fu_24_ and *Pf*MSP-Fu_24_-loaded elastic liposomal formulations. The statistical data areexpressed as mean ± SE (n = 4 or 6). (* *p* < 0.05, ** *p* < 0.01, and *** *p* < 0.001). Table S1: Primers sequence used for qRT-PCR; Table S2: Estimation of zeta potential for elastic liposomes-loaded *Pf*MSP-Fu_24_ (EL-*Pf*MSP-Fu_24_).
