# Supplementary material for: Transdermal Immunization of Elastic Liposome-Laden Recombinant Chimeric Fusion Protein of P. falciparum (PfMSP-Fu24) Mounts Protective Immune Response
Source: Nanomaterials (Basel). 2021 Feb 5;11(2):406. doi: 10.3390/nano11020406 (PMC7914931; doi:10.3390/nano11020406)
Supplement: Supplementary file 1 [file nanomaterials-11-00406-s001.pdf]

Supplementary Figures

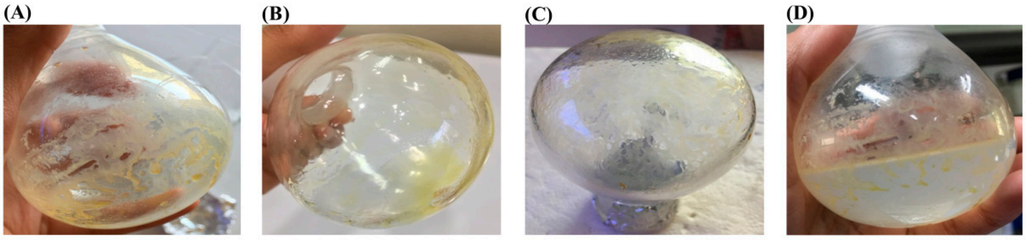

Supplementary Figure 1: (A, B, C) Thin Lipid film formation 86:14 w/w soya lecithin liquid:span-80, 7:3 (v/v) chloroform: methanol **(D)** thin film hydration suspension of lipid film in PBS (pH 6.5) 15µg/ml BSA dissolved in 1x PBS (pH 6.5)

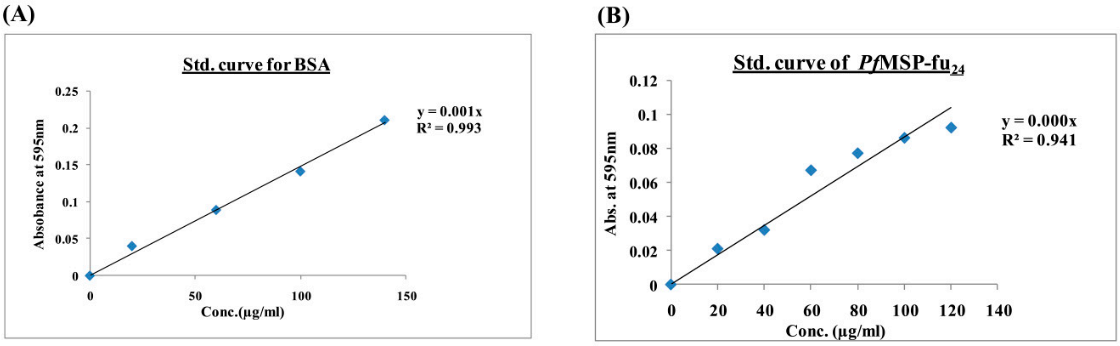

Supplementary Figure 2: Standard calibration curve drawn for, **(A)** BSA and **(B)***Pf*MSP-Fu24 at 595nm wavelength

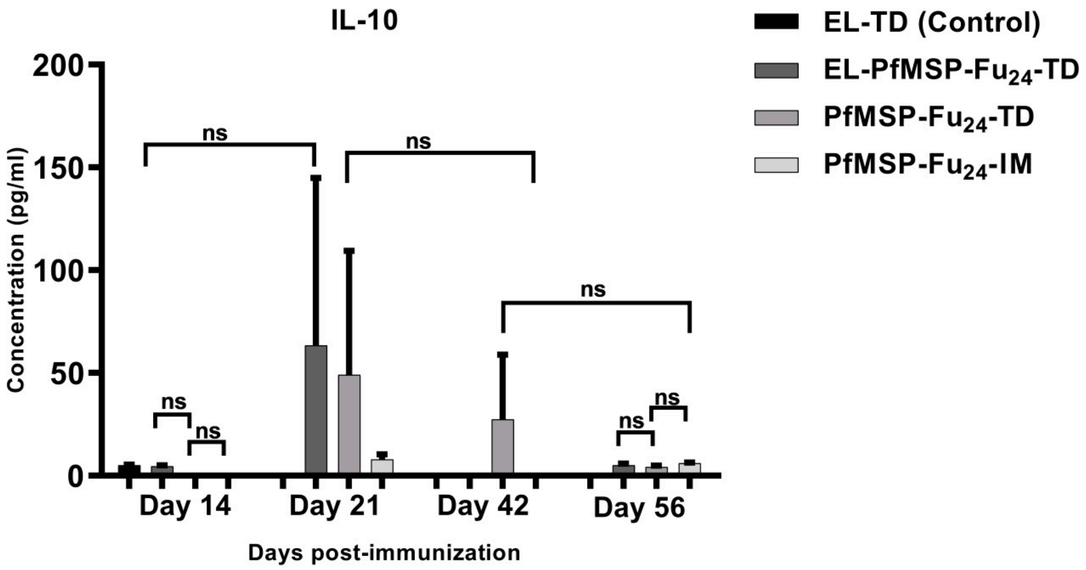

SI Figure 3: Serum quantification of immuno-regulatory cytokine, IL-10 in the mice vaccinated with plain *Pf*MSP-Fu24 and *Pf*MSP-Fu24 loaded elastic liposomal formulations. The statistical data is expressed as mean ± SE (n = 3 or 5). (*p < 0.05, **p < 0.01 and ***p < 0.001)

Supplementary Tables

Supplementary Table 1. Estimation of Zeta potential for elastic liposomes loaded *Pf*MSP-Fu24 (EL- *Pf*MSP-Fu24)

**Summary**		**Percentiles**		**Peak Summary**		
Data	Value	%Tile	Size (nm)	Dia (nm)	Vol%	Width
MI(nm)	605.0	10.00	91.30	1528	25.2	689.00
MN(nm)	64.70	20.00	141.7	238.0	74.8	404.00
MA(nm)	216.8	30.00	194.4	**Zeta Potential**		
CS	27.67	40.00	254.9	**Mobility**	0.73u/s/V/cm	
SD	643.0	50.00	334.0	**Zeta Potential**	9.36 mv	
PDI	1.990	60.00	456.0	**Charge**	0.049 fC	
Mz	620.9	70.00	696.0	**Polarity**	Positive	
Ski	680.8	80.00	1232	**Conductivity**	6,662 uS/cm	
Kg	924.7	90.00	1620	**Concentration**	1	
		95.00	1815	**Dielectric Const**	79	

Supplementary Table 2. Primer sequence used for qRT-PCR

**Cytokine gene**	**Primer Sequences**
GAPDH	F: 5-TTCACCACCATGGAGAAGGC-3<br>R: 5-GGCATGGACTGTGGTCATGA-3
IL-12β	F: 5-GGAAGCACGGCAGCAGAATA-3<br>R: 5-AACTTGAGGGAGAAGTAGGAAG-3
IFN-γ	F: 5-TCAAGTGGCATAGATGTGGAAGAA -3<br>R: 5-TGGCTCTGCAGGATTTTCATG-3
TNF-α	F: 5-TCTCAGCCTCTTCTCATTCC-3<br>R: 5-CGATCACCCCGAAGTTC-3
TGF- β	F: 5-CTATGCTAAAGAGGTCACCCG-3<br>R: 5-ACTGCTTCCCGAATGTCTG-3

F indicates 'Forward primer' and R indicates 'Reverse primer'
